# Stereomicroscopic evaluation of dentinal microcracks after instrumentation with rotary and reciprocating file systems: An *in vitro* observational study

**DOI:** 10.6026/973206300213814

**Published:** 2025-10-31

**Authors:** Vaishnavi Gupta, Rahul Pandey, Manoj Kumar Hans, Nirma Bharati, Archita Goel, Palsodkar Aishwarya

**Affiliations:** 1 Department of Conservative Dentistry and Endodontics, Institute of Dental Sciences, Bareilly, Uttar Pradesh, India

**Keywords:** Dentinal microcracks, endodontics, reciprocating files, rotary files, root canal preparation, stereomicroscopy

## Abstract

Root canal instrumentation was associated with dentinal microcrack formation in all groups, with no statistically significant differences observed. The single-file reciprocating system showed fewer apical microcracks, while the rotary system demonstrated more apical defects but performed better at the coronal level. Complete fractures were relatively uncommon but occurred more frequently with reciprocating files at the coronal level. These variations suggest that factors beyond instrumentation kinematics, including file design and metallurgy, contribute to microcrack development. Further studies with advanced imaging are needed to validate these findings and assess their clinical implications.

## Background:

Endodontic therapy aims to eliminate microorganisms, necrotic tissue and debris from the root canal system while creating morphology conducive to optimal disinfection and hermetic seal [1]. During the cleaning and shaping process, mechanical stress is applied to the root canal walls, which may lead to the development of dentinal defects such as craze lines, incomplete cracks, or complete fractures [2]. These microstructural changes can compromise the long-term prognosis of endodontically treated teeth, as they may progress to vertical root fractures under occlusal loading [3]. Vertical root fractures represent a significant clinical challenge, often necessitating tooth extraction [4]. These fractures frequently originate from minor structural defects in the dentin that can propagate over time [5]. The extent and nature of such damage largely depend on the instrumentation technique and file system used during canal preparation [6]. Continuous stress, particularly from occlusal forces, can exacerbate these microcracks, increasing the risk of complete root fracture [7]. Nickel-titanium (NiTi) rotary instrumentation systems have gained widespread popularity over the past few decades due to their superior flexibility, resistance to torsional and flexural fatigue, and efficient cutting ability [8]. However, continuous rotary motion can exert considerable torsional and lateral stresses on canal walls, potentially leading to an increased risk of microcrack formation [9]. These limitations have encouraged the development of reciprocating systems that utilize alternating clockwise and counterclockwise motions [10].

Reciprocating files function using a repetitive oscillatory movement, initially rotating in a counterclockwise direction to cut dentin, then reversing in a clockwise direction to disengage, helping to minimize the screw-in effect often seen with continuously rotating systems [11]. This motion is supported by a lateral brushing action, which enhances the efficiency of root canal shaping while reducing stress on the instrument [12]. The concept behind these systems is that the file engages and disengages with the root canal wall quickly and repeatedly, reducing torsional stress and improving safety [13]. Several methods have been employed to evaluate microcracks in root canal dentin, including destructive techniques such as stereomicroscopy and scanning electron microscopy (SEM), and non-destructive methods like micro-computed tomography (micro-CT) [14]. Stereomicroscopy remains one of the most popular methods due to its accessibility and ability to provide three-dimensional evaluation of dental structures [15]. Despite numerous studies comparing different file systems, the frequency of dentinal cracks in newer rotary and reciprocating file systems has not been thoroughly assessed and compared [16]. Therefore, it is of interest to compare the incidence of dentinal microcracks in root dentin following endodontic instrumentation using rotary and reciprocating file systems through stereomicroscopic evaluation.

## Materials and Methods:

## Study design and sample size:

This *in vitro* observational study was conducted at the Department of Conservative Dentistry and Endodontics and the Department of Oral Pathology and Microbiology, Institute of Dental Sciences, Bareilly (U.P), over a period of one year (May 2023 - April 2024). The sample size was determined using G*power software version 3.0.1 (Franz Faul, Universität Kiel, Germany). Based on an effect size of 0.4, α error probability of 0.05, power (1-β error probability) of 0.9, and three groups, a total sample size of 84 teeth was calculated (28 per group).

## Sample selection and preparation:

Freshly extracted mandibular premolar teeth were collected and stored in normal saline until use. Teeth were cleaned using an ultrasonic scaler to remove any plaque or calculus. Inclusion criteria comprised extracted mandibular premolars with sound tooth structure, proper anatomic morphology, and completely formed roots. Exclusion criteria included teeth with dental caries, previous endodontic treatment, dental prostheses, metallic restorations, or fractures. All teeth were decoronated using a low-speed saw with coolant to achieve a standardized root length of 16mm. Each root was covered with aluminum foil and embedded in acrylic resin within an acrylic tube. After polymerization, the root was removed, and the foil was replaced with a hydrophilic vinyl polyvinyl siloxane impression material to simulate the periodontal ligament. The root was then immediately repositioned in the acrylic block. To prevent dehydration, the apical 3mm of each root was left uncovered and dipped in water throughout the experimental procedures.

## Group allocation and instrumentation:

The teeth were randomly divided into three groups (n=28):

[1] Group A: Rotary file system (ProTaper Next, Dentsply Maillefer, Ballaigues, Switzerland)

[2] Group B: Multiple-file reciprocating system (Reciproc Blue, VDW, Munich, Germany)

[3] Group C: Single-file reciprocating system (WaveOne Gold, Dentsply Maillefer, Ballaigues, Switzerland)

Access opening was performed using an air-rotor handpiece and an Endo access bur. Canal patency was checked with a #15 K-file (Dentsply Maillefer), and a glide path was prepared up to #20. Sodium hypochlorite (1.5%) solution was used as an irrigant throughout the instrumentation, followed by a final rinse with normal saline. Groups A, B, and C were prepared according to the manufacturers' instructions for their respective file systems. All instrumentation procedures were performed by a single operator with expertise in endodontics to ensure consistency.

## Sectioning and evaluation:

Following root canal preparation, the teeth in all groups were rinsed with distilled water and sectioned perpendicular to the long axis at 3, 6, and 9mm from the apex using a low-speed diamond disc under water cooling. Samples were then examined under a stereomicroscope (Olympus SZX7, Tokyo, Japan) at 20x magnification, and images were captured using a attached digital camera. Each specimen was evaluated for the presence or absence of dentinal defects by two independent examiners who were blinded to the group allocation. In case of disagreement, a third examiner was consulted for final decision.

Fractures were classified into four types according to established criteria:

[1] No fracture: no dentinal defect or craze lines present, both on external and internal walls of the root

[2] Partial fracture: craze line propagating from the canal lumen toward the outer surface but not reaching it

[3] Complete fracture: fracture line extending completely from the canal lumen to the external surface

[4] Other fracture: microcrack propagating from the outer surface of the root toward the canal lumen but not reaching it

## Statistical analysis:

Data were entered into an Excel spreadsheet and analyzed using SPSS version 24 (IBM Corp., Armonk, NY, USA). Descriptive statistics including mean, standard deviation, and percentages were calculated. The Chi-square test was used to analyze the association between categorical variables. A two-sided p-value <0.05 was considered statistically significant. Inter-examiner reliability was assessed using Cohen's kappa coefficient.

## Results:

At the 3mm level, Group C (single-file reciprocating system) demonstrated the highest percentage of samples without fractures (96.4%), followed by Group B (multiple-file reciprocating system) with 85.7%, and Group A (rotary file system) with 75.0%. Complete fractures were observed only in Group A (3.6%), while partial fractures were also limited to Group A (7.1%). Other fractures were present in 14.3% of samples in both Groups A and B, but only in 3.6% of samples in Group C. However, these differences were not statistically significant (p>0.05) (Table 1 - see PDF). At the 6mm level, all three groups showed similar percentages of "no fracture" cases: 75.0% in Groups A and C, and 78.6% in Group B. Complete fractures were present in all groups, with Groups A and C showing 7.1% each, and Group B showing 3.6%. Partial fractures appeared only in Group B (3.6%). Other fractures were most common in Groups A and C (17.9% each), compared to Group B (14.3%). No statistically significant differences were found among the groups at this level (p>0.05) (Table 2 - see PDF). At the 9mm level, Group A (rotary file system) had the highest percentage of intact samples (82.1%), followed closely by Groups B and C (both 78.6%). Interestingly, Group C showed the highest incidence of complete fractures (10.7%), compared to Groups A and B (3.6% each). Partial fractures were observed only in Group B (3.6%). Other fractures were present in 14.3% of samples in Group A, 14.3% in Group B, and 10.7% in Group C. These differences were not statistically significant (p>0.05) (Table 3 - see PDF). The inter-examiner reliability for the assessment of dentinal defects was excellent, with a Cohen's kappa coefficient of 0.89, indicating high agreement between the examiners.

## Discussion:

The present study aimed to evaluate and compare the incidence of dentinal microcracks following root canal preparation using rotary and reciprocating file systems. The findings revealed that while all instrumentation systems produced some dentinal defects, the single-file reciprocating system (Group C) demonstrated the least number of microcracks at the apical level (3mm), whereas the rotary file system (Group A) showed the highest incidence of defects at this level. However, these differences were not statistically significant. At the 3mm level, Group C showed 96.4% of samples without fractures, compared to 85.7% in Group B and 75.0% in Group A. This finding is consistent with previous studies suggesting that reciprocating motion generates less stress on dentinal walls compared to continuous rotation [17]. The superior performance of the single-file reciprocating system may be attributed to its reciprocating motion, which has been shown to reduce torsional stress on the canal walls [18]. Additionally, the single-file usage reduces the accumulation of repeated stress, potentially minimizing the risk of microcrack formation [19]. Interestingly, at the 6mm level, all three groups showed similar percentages of "no fracture" cases, ranging from 75.0% to 78.6%. This suggests that the middle third of the root may be equally susceptible to instrument-induced stress regardless of the instrumentation technique used [20]. The complex anatomy and higher canal curvature in this region might contribute to the similar incidence of defects across all groups [21]. At the 9mm level, Group A (rotary file system) demonstrated the highest percentage of intact samples (82.1%), while Groups B and C showed 78.6% each. This finding contradicts the initial expectation that reciprocating systems would consistently produce fewer microcracks. The higher incidence of complete fractures in Group C at this level (10.7%) might be attributed to the interaction between reciprocating motion and the wider canal diameter in the coronal third, resulting in elevated lateral stress on the dentinal walls [22, 23- 24]. reported that reciprocating files caused less dentinal damage than rotary files due to reduced torsional stress. However, our findings at the coronal level differ from these studies, highlighting the complex relationship between instrumentation kinematics and dentinal microcrack formation. It is important to note that the method used for evaluating dentinal defects in this study (stereomicroscopy) has certain limitations. Sectioning the roots may introduce artificial cracks, potentially affecting the results [25]. Micro-computed tomography (micro-CT) has been suggested as a more accurate method for detecting true microcracks without introducing artifacts [26]. However, stereomicroscopy remains a valuable tool due to its accessibility and ability to provide direct visualization of dentinal defects [27]. The null hypothesis of this study was accepted as no statistically significant differences were found among the three groups. This suggests that instrumentation kinematics alone may not be the primary determinant of dentinal microcrack formation. Instead, multiple factors such as file tip configuration, cross-sectional shape, taper, and the metallurgical characteristics of the NiTi alloy may collectively contribute to the occurrence of dentinal defects [28]. The clinical implications of these findings are significant. Dentinal microcracks may propagate over time, potentially leading to vertical root fractures and tooth loss [29]. While the differences among the systems were not statistically significant, the trend toward fewer microcracks with reciprocating systems in the apical region suggests that these systems may be advantageous in preserving tooth structure [30]. However, the choice of instrumentation system should be based on a comprehensive evaluation of multiple factors, including canal anatomy, operator experience, and the specific clinical situation [31].

## Conclusion:

All tested instrumentation systems were associated with dentinal microcrack formation, with no significant overall differences between them. Variations observed at apical and coronal levels suggest that factors beyond kinematics, such as file design and material properties, may influence outcomes. Further studies with advanced imaging are needed to better understand the clinical relevance of these findings.
